# Effect of Polydispersity on the Structural and Magnetic Properties of a Magnetopolymer Composite

**DOI:** 10.3390/polym15122678

**Published:** 2023-06-14

**Authors:** Dmitry I. Radushnov, Anna Yu. Solovyova, Ekaterina A. Elfimova

**Affiliations:** Institute of Natural Sciences and Mathematics, Ural Federal University, 51 Lenin Avenue, Ekaterinburg 620000, Russia; dmitry.radushnov@urfu.ru (D.I.R.); anna.soloveva@urfu.ru (A.Y.S.)

**Keywords:** magnetopolymer composite, polydisperse magnetic filler, magnetization, orientational anisotropy, dipole–dipole interaction, bidisperse model

## Abstract

When using magnetopolymer composites in high-precision industrial and biomedical technologies, the problem of predicting their properties in an external magnetic field arises. In this work, we study theoretically the influence of the polydispersity of a magnetic filler on a composite’s equilibrium magnetization and on the orientational texturing of magnetic particles formed during polymerization. The results are obtained using rigorous methods of statistical mechanics and Monte Carlo computer simulations in the framework the bidisperse approximation. It is shown that by adjusting the dispersione composition of the magnetic filler and the intensity of the magnetic field at which the sample’s polymerization occurs, it is possible to control the composite’s structure and magnetization. The derived analytical expressions determine these regularities. The developed theory takes into account dipole–dipole interparticle interactions and therefore can be applied to predict the properties of concentrated composites. The obtained results are a theoretical basis for the synthesis of magnetopolymer composites with a predetermined structure and magnetic properties.

## 1. Introduction

Magnetopolymer composites (MPC) consist of fine magnetic particles embedded in polymer matrices [1,2,3]. These materials belong to the class of smart materials because of their high response to magnetic fields, which makes it possible to control their behavior and properties. The response of a magnetic filler to an applied magnetic field is determined by two main physical mechanisms: Brownian and Néele rotation of the magnetic moments. Brownian relaxation is characterized by a change in the direction of the magnetic moment along with the rotation of the particle, while during the Néel relaxation, the magnetic moment rotates inside the particle’s body. For ensembles of magnetic particles suspended in liquid carriers, both mechanisms take place. When particles are embedded into a polymer matrix, they lose their translational and orientational degrees of freedom. In this case, Néel relaxation becomes the main mechanism determining the magnetic properties of ensembles of such immobilized particles. Modern methods of MPC synthesis offer various mechanisms for embedding magnetic particles into a polymer matrix [4,5,6,7,8,9,10]. One is the addition of magnetic particles into a liquid polymer solution, followed by curing of the medium [11,12,13,14,15]. The magnetic field acting on the dispersion of particles forms the orientation and structural texturing of the magnetic filler, which can be “frozen” during polymerization. As a result, the MPC polymer matrix maintains the position of the particles and the direction of their easy magnetization axes was generated before polymerization. The specificity of the magnetic filler’s internal structure in the MPC can be controlled via field intensity, temperature, concentration of nanoparticles, dispersion composition, and the intensity of dipole–dipole interactions. All these factors are control parameters in the synthesis of the MPC, which can regulate the properties of the MPC and its response to the magnetic field.

The dependence of the MPC’s properties on the structuring features of the magnetic filler has been demonstrated in experimental works [9,16,17,18,19]. To predict the magnetic properties of an MPC, theories have been developed for various cases of the magnetic filler’s orientation texturing: the easy magnetization axes are aligned parallel to each other [20,21,22,23,24,25,26,27,28], orientation texturing is absent [27,29,30,31], and the orientation of the easy magnetization axes is formed under the influence of a constant magnetic field [32,33,34]. A magnetic response theory of immobilized particles with a specific spatial arrangement of magnetic particles has been developed in [35,36,37,38,39]. Theoretical works are usually based on monodisperse models, assuming that all magnetic filler particle have the same size. However, it is well known that the magnetic filler is polydisperse, which significantly affects the sample’s properties [40,41].

In this article, the influence of polydispersity on the orientation architecture of an MPC magnetic filler formed during synthesis is theoretically studied; the magnetization of the polydisperse MPC in a static magnetic field is investigated. The theory takes into account dipole–dipole interparticle interactions, so it can be used to predict the properties of concentrated MPCs. As has been shown in [42,43,44,45], the effects of polydispersity observed in experiments can be successfully described at the level of bidisperse (BD) theory, so here we will focus on the BD model. The theory developed in the article is the basis for the synthesis of an MPC with a predefined internal architecture and a predictable response to a static magnetic field.

## 2. Bidisperse Model

**Figure 1:**
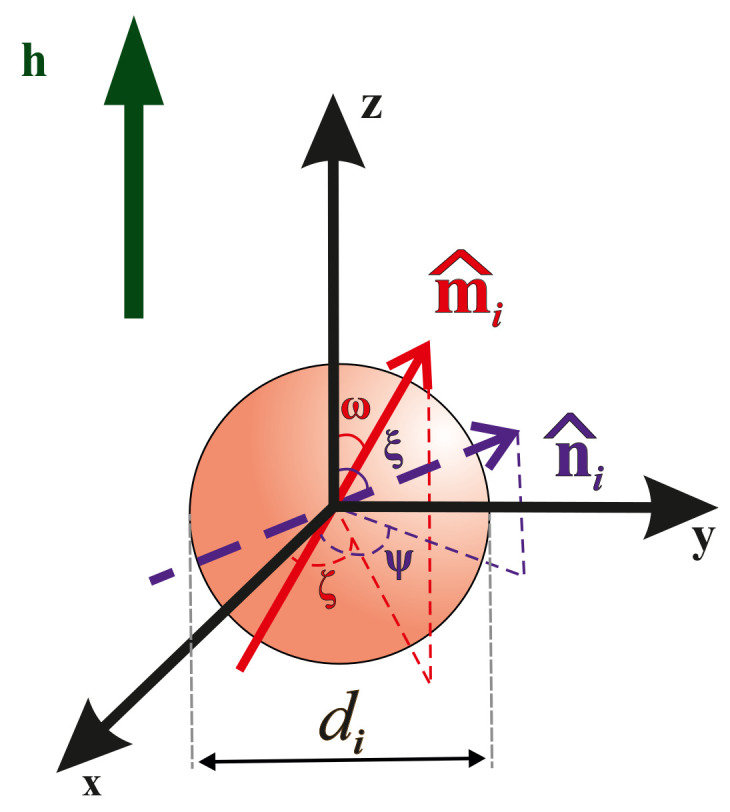
Model of a particle contained in a magnetic filler.

We consider a BD system of uniformly magnetized hard spherical particles. The system’s two components we will call small and large fractions. The small fraction contains $N_{s}$ particles with diameter $d_{s}$; the large fraction consists of $N_{l}$ particles with diameter $d_{l}$. The particles are assumed to be distributed throughout a long, cylindrical tube with volume *V* at temperature *T*. The height of the tube is oriented along the laboratory Oz axis, and the applied magnetic field $h=h\hat{h}=h\left(0,\;0,\;1\right)$ is in the same direction. This means that demagnetization effects can be neglected, while the internal magnetic field can be taken to be the same as the external applied field h. The magnetic material of the particles is characterized by bulk saturation magnetization $M_{0}$ and the magnetic anisotropy constant *K*. The magnetic moment of *i*-th particle is $m_{i}=M_{0}\pi d_{i}^{3}/6$. We assume that the particle material has a uniaxial magneto-crystalline structure: therefore, the direction of the particle’s easy magnetization axis is defined by unit vector ${\hat{n}}_{i}=\left(\sin\xi_{i}\cos\psi_{i},\sin\xi_{i}\sin\psi_{i},\cos\xi_{i}\right)$ in spherical coordinate system. Due to Néel superparamagnetizm the magnetic moments can rotate inside the particles. So, the direction of *i*-th magnetic moment $m_{i}=m_{i}{\hat{m}}_{i}=m_{i}\left(\sin\omega_{i}\cos\zeta_{i},\sin\omega_{i}\sin\zeta_{i},\cos\omega_{i}\right)$ differs from ${\hat{n}}_{i}$ (Figure 1). The center position of each *i*-th particle is defined by its radius-vector $r_{i}=r_{i}\left(\sin\theta_{i}\cos\phi_{i},\sin\theta_{i}\sin\phi_{i},\cos\theta_{i}\right)$. The particle number concentration is ρ=N/V, where the parameter $N=N_{s}+N_{l}$ is the total number of ferroparticles. The total volume fraction φ is the sum of the volume fractions of the small and large particles: $\varphi =\varphi_{s}+\varphi_{l}$, $\varphi_{s}=N_{s}\pi d_{s}^{3}/6V$, and $\varphi_{s}=N_{l}\pi d_{l}^{3}/6V$.

The system’s total potential energy *U* contains the contributions from the pair interparticle dipole–dipole interactions $U_{d}\left(ij\right)$, the single particle interactions of particle dipoles with the external magnetic field $U_{m}\left(i\right)$, and the internal magnetic anisotropy potential $U_{\sigma }\left(i\right)$. Thus, for a configuration of *N* particles, the energy *U* can be written as:

(1) $$U={\hat{H}}_{d}+{\hat{H}}_{m}+{\hat{H}}_{\sigma },$$

(2) $${\hat{H}}_{d}=\sum_{i<j=1}^{N}U_{d}\left(ij\right),\;U_{d}\left(ij\right)=\frac{\mu_{0}m_{i}m_{j}}{4\pi r_{ij}^{3}}\left[\left({\hat{m}}_{i}\cdot {\hat{m}}_{j}\right)-3\left({\hat{m}}_{i}\cdot {\hat{r}}_{ij}\right)\left(\hat{m_{j}}\cdot {\hat{r}}_{ij}\right)\right],$$

(3) $${\hat{H}}_{m}=\sum_{i=1}^{N}U_{m}\left(i\right),\;U_{m}\left(i\right)=-\mu_{0}m_{i}h\left({\hat{m}}_{i}\cdot \hat{h}\right),$$

(4) $${\hat{H}}_{\sigma }=\sum_{i=1}^{N}U_{\sigma }\left(i\right),\;U_{\sigma }\left(i\right)=-K\frac{\pi d_{i}^{3}}{6}{\left({\hat{m}}_{i}\cdot {\hat{n}}_{i}\right)}^{2},$$

where $\mu_{0}$ is the vacuum magnetic permeability, $r_{ij}=r_{i}-r_{j}=r_{ij}{\hat{r}}_{ij}$ and ${\hat{r}}_{ij}$ denotes the unit vector of $r_{ij}$.

There are several dimensionless parameters that measure the corresponding energies with respect to the thermal energy $k_{B}T$:

(5) $$\alpha_{i}=\frac{\mu_{0}m_{i}h}{k_{B}T}\;,\;\sigma_{i}=\frac{K\pi d_{i}^{3}}{6k_{B}T},\;\lambda_{ij}=\frac{2\mu_{0}m_{i}m_{j}}{\pi k_{B}T{\left(d_{i}+d_{j}\right)}^{3}},$$

where $\alpha_{i}$ is the Langevin parameter characterizing the dipole-field interactions, $\sigma_{i}$ is the magneto-crystalline anisotropy parameter, and $\lambda_{ij}$ is dipolar coupling constant, which determines the ratio of the magnetic energy at the contact of particles *i* and *j* with the thermal energy. We will use the notation $\alpha_{s}$, $\sigma_{s}$, and $\lambda_{s}$ if particles *i* and *j* belong to the small particle fraction; $\alpha_{l}$, $\sigma_{l}$, and $\lambda_{l}$ will correspond to interactions for the large particle fraction.

The process being modeled can be divided into two steps. In the first step, the sample’s orientational structure is formed: in response to a magnetic field, particles move and rotate, changing the orientation of their magnetic moments and easy magnetization axes. Once thermodynamic equilibrium is reached, the orientational and rotational degrees of freedom of the particles are fixed. As a result, an orientational texture of the easy axes is formed in the ensemble of immobilized particles. In the second step, the ensemble of immobilized particles is placed in a magnetic field directed parallel to the field that was applied during the first step. Due to Néel relaxation, the magnetic moments of the particles change their orientation, resulting in the sample’s magnetization. We assume that both steps take place at the same temperature *T*, so that the magnetic anisotropy constant $\sigma_{i}$ and the dipole–dipole interaction parameter $\lambda_{ij}$ have the same values at each step of the process. In the first step, the magnetic field $h^{p}$ with the intensity $h^{p}$ is directed parallel to the axis Oz; the magnetic field h acting on the system in the second step has the intensity *h* and $h||h^{p}$. Consequently, the Langevin parameter characterising the magnetic field intensity takes different values in the first and second steps of the process; they will be denoted by $\alpha_{i}$ and $\alpha_{i}^{p}$, respectively.

## 3. Bidisperse Theory: Structural and Magnetic Properties

It is assumed that the small fraction includes particles with numbers from 1 to *k*, and the large fraction includes particles with numbers k+1…N. The two-particle distribution function $W_{0}\left({\hat{r}}_{1},{\hat{n}}_{1},{\hat{r}}_{k+1},{\hat{n}}_{k+1}\right)$ of an ensemble of moving particles in the field $h^{p}$ has the meaning of the probability density that a randomly chosen small particle with number 1 and a randomly chosen large particle with number k+1 have directions of the easy magnetization axes ${\hat{n}}_{1},{\hat{n}}_{k+1}$, and their positions are described by the vectors ${\hat{r}}_{1},{\hat{r}}_{k+1}$, respectively:

(6) $$W_{0}\left({\hat{r}}_{1},{\hat{n}}_{1},{\hat{r}}_{k+1},{\hat{n}}_{k+1}\right)=\frac{1}{Z_{0}}\int \int \int \exp\left[\frac{{\hat{H}}_{\sigma }+{\hat{H}}_{m}^{p}+{\hat{H}}_{d}}{k_{B}T}\right]d{\hat{n}}_{2}\ldots d{\hat{n}}_{k}d{\hat{n}}_{k+2}\ldots d{\hat{n}}_{N}d\hat{m}d{\hat{r}}_{2}\ldots d{\hat{r}}_{k}d{\hat{r}}_{k+2}\ldots d{\hat{r}}_{N},$$

$$Z_{0}=\int \int \int \exp\left[\frac{{\hat{H}}_{\sigma }+{\hat{H}}_{m}^{p}+{\hat{H}}_{d}}{k_{B}T}\right]d\hat{n}d\hat{m}d\hat{r},$$

where the superscript *p* in the Hamiltonian ${\hat{H}}_{m}^{p}$ denotes that the sample is placed in the field $h^{p}$; $\hat{n}=\left\{{\hat{n}}_{1}\ldots {\hat{n}}_{N}\right\}$, $\hat{m}=\left\{{\hat{m}}_{1}\ldots {\hat{m}}_{N}\right\}$, $\hat{r}=\left\{{\hat{r}}_{1}\ldots {\hat{r}}_{N}\right\}$, and the averaging over the freedom degrees of particle *i* is determined by the formulas

(7) $$\int d{\hat{m}}_{i}=\frac{1}{4\pi }\int_{0}^{2\pi }d\zeta_{i}\int_{-1}^{1}d\cos\omega_{i},\;\int d{\hat{m}}_{i}\cdot 1=1,$$

(8) $$\int d{\hat{n}}_{i}=\frac{1}{4\pi }\int_{0}^{2\pi }d\psi_{i}\int_{-1}^{1}d\cos\xi_{i},\;\int d{\hat{n}}_{i}\cdot 1=1,$$

(9) $$\int d{\hat{r}}_{i}=\frac{1}{V}\lim_{R\to \infty }\int_{0}^{2\pi }d\phi_{i}\int_{-1}^{1}d\cos\theta_{i}\int_{0}^{R/\sin\theta_{i}}r_{i}^{2}dr_{i},\;\int d{\hat{r}}_{i}\cdot 1=1.$$

Under conditions of thermodynamic equilibrium, the orientation texture of a BD system of moving particles can be described by a one-particle distribution function of easy magnetization axes

(10) $$f^{p}\left(\hat{n}\right)=\nu_{s}\int \int W_{0}\left({\hat{r}}_{1},\hat{n},{\hat{r}}_{k+1},{\hat{n}}_{k+1}\right)d{\hat{r}}_{1}d{\hat{r}}_{k+1}d{\hat{n}}_{k+1}+\nu_{l}\int \int W_{0}\left({\hat{r}}_{1},{\hat{n}}_{1},{\hat{r}}_{k+1},\hat{n}\right)d{\hat{r}}_{1}d{\hat{r}}_{k+1}d{\hat{n}}_{1},$$

where $\nu_{s}=N_{s}/N$ and $\nu_{l}=N_{l}/N$ are the fractions of small and large particles, respectively. The integrals in the first and second terms on the right-hand side of Equation (10) determine the probability density of the orientation of the easy axis of random small and large particles in direction $\hat{n}$, respectively. Since the system of immobilized particles is formed from a thermodynamic equilibrium ensemble of moving particles by fixing the location of the particles and the orientation of their easy axes, function $f^{p}\left(\hat{n}\right)$ will also determine the internal structure of the ensemble of immobile particles. Restricting the expansion of the Hamiltonians to terms of the first order in the Langevin susceptibility $\chi_{L}$ and averaging over all values of the azimuth angle ψ of vector $\hat{n}$, the explicit analytic expression for $f^{p}\left(\xi \right)$ as a function of the polar angle ξ of vector $\hat{n}$ can be written as

(11) $$f^{p}\left(\xi \right)=\nu_{s}f_{sl}^{p}\left(\xi \right)+\nu_{l}f_{ls}^{p}\left(\xi \right),$$

(12) $$f_{ab}^{p}\left(\xi \right)=\frac{P(\alpha_{a}^{p},\sigma ,\xi )}{R(\alpha_{a}^{p},\sigma )}+\frac{1}{2R(\alpha_{a}^{p},\sigma )}\left[\frac{\chi_{L}^{aa}G\left(\alpha_{a}^{p},\sigma \right)}{R(\alpha_{a}^{p},\sigma )}+\frac{\chi_{L}^{ab}G\left(\alpha_{b}^{p},\sigma \right)}{R(\alpha_{b}^{p},\sigma )}\right]\left[\frac{\partial P(\alpha_{a}^{p},\sigma ,\xi )}{\partial \alpha_{a}^{p}}-\frac{G\left(\alpha_{a}^{p},\sigma \right)P\left(\alpha_{a}^{p},\sigma ,\xi \right)}{R(\alpha_{a}^{p},\sigma )}\right],$$

$$P\left(\alpha ,\sigma ,\xi \right)=\frac{1}{2}\int_{-1}^{1}\exp\left(\sigma t^{2}+\alpha t\cos\xi \right)I_{0}\left(\alpha \sqrt{1-t^{2}}\sin\xi \right)dt,$$

$$R\left(\alpha ,\sigma \right)=\left(\frac{\sinh\alpha }{\alpha }\right)\int_{0}^{1}\exp\left(\sigma t^{2}\right)dt,\;G\left(\alpha ,\sigma \right)=\frac{1}{2}\int_{0}^{\pi }\frac{\partial P(\alpha ,\sigma ,\xi )}{\partial \alpha }\sin\xi d\xi ,$$

where $\chi_{L}^{ab}$ is the fractional Langevin parameter, which is determined by the formulas

$$\chi_{L}^{ss}=\frac{\mu_{0}\rho_{s}m_{s}^{2}}{3k_{B}T},\;\chi_{L}^{ll}=\frac{\mu_{0}\rho_{l}m_{l}^{2}}{3k_{B}T},\;\chi_{L}^{sl}=\frac{\mu_{0}\left(\rho_{s}+\rho_{l}\right)m_{s}m_{l}}{3k_{B}T}.$$

The method of expanding Hamiltonians into a series in terms of the Langevin susceptibility $\chi_{L}$ is described in detail in Ref. [33] for a monodisperse ensemble of magnetic particles. It is worth noting that if we replace $d_{s}=d_{l}$, $\nu_{s}=1$, $\nu_{l}=0$ (or $d_{l}=d_{s}$, $\nu_{s}=0$, $\nu_{l}=1$), then Equations (11) and (12) are exactly transformed into the expression for the magnetic moment orientation probability density of a monodisperse system derived in [33] (Equation (14) in [33]). In Equations (11) and (12), the terms of order $∼\chi_{L}^{ij}$ determine the contribution of the dipole–dipole interaction to the distribution function of easy axes;the zero order terms in the Langevin susceptibility correspond to the ideal system approximation:

(13) $$f^{p(id)}\left(\xi \right)=\nu_{s}\frac{P(\alpha_{s}^{p},\sigma ,\xi )}{R(\alpha_{s}^{p},\sigma )}+\nu_{l}\frac{P(\alpha_{l}^{p},\sigma ,\xi )}{R(\alpha_{l}^{p},\sigma )}.$$

The probability density (13) coincides with the monodisperse one-particle theory [32] (Equation (6) in [32]) when the fraction of small $\nu_{s}$ or large $\nu_{l}$ particles in the system becomes zero.

The degree of the alignment of the particle easy axes can be described with the help of the second moment $Q_{2}$:

(14) $$Q_{2}=\frac{1}{2}\int_{0}^{\pi }f^{p}\left(\xi \right)\frac{3\cos^{2}\xi -1}{2}\sin\xi d\xi .$$

The second moment $Q_{2}$ characterizes the ordering of the easy axes in the system: it is zero for a uniform random configuration and equal to unity for complete parallel alignment.

In the applied magnetic field h the particle magnetic moments tend to turn in the field’s direction. At this point in the ensemble of immobilized particles with a distribution function $W_{0}\left({\hat{r}}_{1},{\hat{n}}_{1},{\hat{r}}_{k+1},{\hat{n}}_{k+1}\right)$ the change in magnetic moment orientation occurs inside the particle body according to the Néel mechanism, since the rotational and translational degrees of freedom of the particles are fixed. In such a system, the probability density that a randomly chosen small particle with number 1 and a randomly chosen large particle with number k+1 have magnetic moment directions ${\hat{m}}_{1},{\hat{m}}_{k+1}$ and easy axes ${\hat{n}}_{1},{\hat{n}}_{k+1}$; their positions, described by vectors ${\hat{r}}_{1},{\hat{r}}_{k+1}$, respectively, can be defined as follows:

$$W\left({\hat{n}}_{1},{\hat{m}}_{1},{\hat{r}}_{1},{\hat{n}}_{k+1},{\hat{m}}_{k+1},{\hat{r}}_{k+1}\right)=W_{0}\left({\hat{r}}_{1},{\hat{n}}_{1},{\hat{r}}_{k+1},{\hat{n}}_{k+1}\right)\int \int \int \exp\left[\frac{{\hat{H}}_{m}+{\hat{H}}_{\sigma }+{\hat{H}}_{d}}{k_{B}T}\right]$$

$$\times \frac{1}{Z_{1}\left(\hat{n},\hat{r}\right)}d{\hat{n}}_{2}\ldots d{\hat{n}}_{k}d{\hat{n}}_{k+2}\ldots d{\hat{n}}_{N}d{\hat{m}}_{2}\ldots d{\hat{m}}_{k}d{\hat{m}}_{k+2}\ldots d{\hat{m}}_{N}d{\hat{r}}_{2}\ldots d{\hat{r}}_{k}d{\hat{r}}_{k+2}\ldots d{\hat{r}}_{N},$$

$$Z_{1}\left(\hat{n},\hat{r}\right)=\int \exp\left[\frac{{\hat{H}}_{m}+{\hat{H}}_{\sigma }+{\hat{H}}_{d}}{k_{B}T}\right]d\hat{m}.$$

The normalization coefficient $Z_{1}\left(\hat{n},\hat{r}\right)$ is chosen so that averaging over all possible orientations of the magnetic moments ${\hat{m}}_{1}$ and ${\hat{m}}_{k+1}$ recovers the structure $W_{0}\left({\hat{r}}_{1},{\hat{n}}_{1},{\hat{r}}_{k+1},{\hat{n}}_{k+1}\right)$ generated during the first step of the process:

$$\int W\left({\hat{n}}_{1},{\hat{m}}_{1},{\hat{r}}_{1},{\hat{n}}_{k+1},{\hat{m}}_{k+1},{\hat{r}}_{k+1}\right)d{\hat{m}}_{1}d{\hat{m}}_{k+1}=W_{0}\left({\hat{r}}_{1},{\hat{n}}_{1},{\hat{r}}_{k+1},{\hat{n}}_{k+1}\right).$$

Thus, the function $W({\hat{n}}_{1},{\hat{m}}_{1},{\hat{r}}_{1},{\hat{n}}_{k+1},{\hat{m}}_{k+1},{\hat{r}}_{k+1})$ describing the magnetic and orientational structure of the particle ensemble during the second step of the process, “remembers” and “keep” the distribution of particles and the orientation of their easy axes formed during the first step of the process.

The magnetization of a BD system of immobilized particles is defined through a pair distribution function $W({\hat{n}}_{1},{\hat{m}}_{1},{\hat{r}}_{1},{\hat{n}}_{k+1},{\hat{m}}_{k+1},{\hat{r}}_{k+1})$:

$$M=\rho_{s}m_{s}\int \int \int \left({\hat{m}}_{1}\cdot h\right)W\left({\hat{n}}_{1},{\hat{m}}_{1},{\hat{r}}_{1},{\hat{n}}_{k+1},{\hat{m}}_{k+1},{\hat{r}}_{k+1}\right)d{\hat{n}}_{1}d{\hat{m}}_{1}d{\hat{r}}_{1}d{\hat{n}}_{k+1}d{\hat{m}}_{k+1}d{\hat{r}}_{k+1}$$

(15) $$+\rho_{l}m_{l}\int \int \int \left({\hat{m}}_{k+1}\cdot h\right)W\left({\hat{n}}_{1},{\hat{m}}_{1},{\hat{r}}_{1},{\hat{n}}_{k+1},{\hat{m}}_{k+1},{\hat{r}}_{k+1}\right)d{\hat{n}}_{1}d{\hat{m}}_{1}d{\hat{r}}_{1}d{\hat{n}}_{k+1}d{\hat{m}}_{k+1}d{\hat{r}}_{k+1}.$$

In Equation (15), using the virial expansion of the distribution function $W({\hat{n}}_{1},{\hat{m}}_{1},{\hat{r}}_{1},{\hat{n}}_{k+1},{\hat{m}}_{k+1},{\hat{r}}_{k+1})$ up to linear terms $∼\chi_{L}^{ab}$ (a,b∈{s,l}), we obtain the analytical formula for magnetization:

(16) $$M=M_{s,l}+M_{l,s}$$

$$M_{a,b}=\frac{\rho_{a}m_{a}}{R(\alpha_{a}^{p},\sigma_{a})}\left\{\int P\left(\alpha_{a}^{p},\sigma_{a},n_{a}\right)\frac{\partial \ln P(\alpha_{a},\sigma_{a},n_{a})}{\partial \alpha_{a}}dn_{a}\left[1-\frac{\chi_{L}^{aa}G^{2}\left(\alpha_{a}^{p},\sigma_{a}\right)}{2R^{2}\left(\alpha_{a}^{p},\sigma_{a}\right)}-\frac{\chi_{L}^{ab}G\left(\alpha_{a}^{p},\sigma_{a}\right)Q\left(\alpha_{b}^{p},\sigma_{b}\right)}{2R\left(\alpha_{a}^{p},\sigma_{a}\right)R\left(\alpha_{b}^{p},\sigma_{b}\right)}\right]+\left[\frac{\chi_{L}^{aa}G\left(\alpha_{a}^{p},\sigma_{a}\right)}{2R(\alpha_{a}^{p},\sigma_{a})}\right.\right.$$

$$\left.+\frac{\chi_{L}^{ab}G\left(\alpha_{b}^{p},\sigma_{b}\right)}{2R(\alpha_{b}^{p},\sigma_{b})}\right]\int \frac{\partial P(\alpha_{a}^{p},\sigma_{a},n_{a})}{\partial \alpha_{a}^{p}}\frac{\partial \ln P(\alpha_{a},\sigma_{a},n_{a})}{\partial \alpha_{a}}dn_{a}+\frac{1}{2}\left[\frac{\chi_{L}^{ab}}{R(\alpha_{b}^{p},\sigma_{b})}\int P\left(\alpha_{b}^{p},\sigma_{b},n_{b}\right)\frac{\partial \ln P(\alpha_{b},\sigma_{b},n_{b})}{\partial \alpha_{b}}dn_{b}\right.$$

(17) $$\left.\left.+\chi_{L}^{aa}\int \frac{\partial \ln P(\alpha_{a},\sigma_{a},n_{a})}{\partial \alpha_{a}}dn_{a}\right]\left[\int \frac{P(\alpha_{a}^{p},\sigma_{a},n_{a})}{P(\alpha_{a},\sigma_{a},n_{a})}\frac{\partial^{2}P\left(\alpha_{a},\sigma_{a},n_{a}\right)}{\partial {\left(\alpha_{a}\right)}^{2}}dn_{a}-\int P\left(\alpha_{a}^{p},\sigma_{a},n_{a}\right){\left(\frac{\partial \ln P(\alpha_{a},\sigma_{a},n_{a})}{\partial \alpha_{a}}\right)}^{2}dn_{a}\right]\right\}.$$

The expansion of the distribution function to a virial series is described in detail in Ref. [33]. In Equations (16) and (17) the terms that do not contain Langevin susceptibility determine the magnetization of an ideal BD system $M^{\left(id\right)}$; the terms $∼\chi_{L}^{ab}$ describe the contribution of the dipole–dipole interaction to the magnetization of a BD ensemble of immobilized magnetic particles. Expressions (16) and (17) are transformed into the magnetization of the monodisperse system (Equations (26) in [33]), assuming $d_{s}=d_{l}$, $\nu_{s}=1$, $\nu_{l}=0$ (or $d_{l}=d_{s}$, $\nu_{s}=0$, $\nu_{l}=1$).

## 4. Computer Simulation

**Figure 2:**
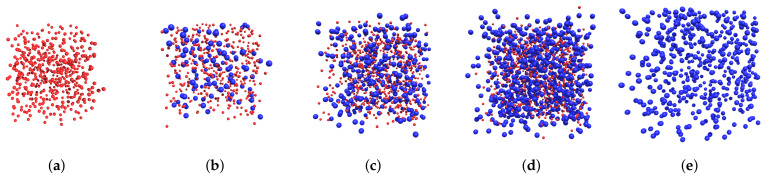
Typical simulation cells for BD configuration with total volume concentrations φ=0.125 in the field $h^{p}=40$ kA/m: (**a**) $\nu_{s}=1$, (**b**) $\nu_{s}=0.8$, (**c**) $\nu_{s}=0.5$, (**d**) $\nu_{s}=0.2$, (**e**) $\nu_{s}=0$.

The BD system was represented by two fractions with magnetic-core diameters $d_{s}=5$ nm and $d_{l}=10$ nm, respectively. Note that the chosen diameters are typical for magnetic particles in magnetopolymer composites, so the model system is able to demonstrate their characteristic behavior. Anisotropy parameters and dipolar coupling constants for each fraction are shown in Table 1. These values correspond to the cobalt ferrite particles with $M_{0}=4.0\times 10^{5}$ A/m and K=200 kJ/m^3^ at room temperature T=293 K [46]. Five different simulation configurations with a total number of particles N=512 and various small particle fractions were studied. The BD systems were simulated at two fixed-volume concentrations, φ=0.125 and φ=0.300, corresponding to saturation magnetizations M(∞)=50 kA/m and M(∞)=120 kA/m, respectively. For each volume concentration and small particle fraction, the Langevin susceptibilities, number of particles, and fraction concentrations are given in Table 2. For each BD configuration with a total volume concentration φ=0.125, two sets of calculations were performed: the magnetization curve $M(h^{p})$ for system of the particles in a liquid carrier before polymerization and the magnetization curve $M(h,h^{p})$ for immobilized particles after polymerization. Typical simulation cells in the field $h^{p}=40$ kA/m are presented in Figure 2. For BD systems with φ=0.300, only the structure properties were studied.

Canonical *NVT* Monte Carlo (MC) simulations were carried out in a cubic simulation box of side *L*, to which 3D periodic boundary conditions were applied. The Ewald summation with conducting boundary conditions was used to calculate the long-range dipole–dipole interactions. Before polymerization, translational and orientational moves of the particles were used in an equiprobable way. Two types of rotation moves were applied: Brownian rotation of both the magnetic moment and easy axis at the same random angle and the Néel rotation of magnetic moment regardless easy axis. For each BD system, $N_{conf}=50$ independent configurations of positions and orientations were saved every $5\times 10^{4}$ attempted translations, while rotations per particle were saved after system equilibration.

For modeling the MPC, the particle positions and easy axes orientations were fixed according to the saved configurations. In this case, MC simulations used Néel rotation in orientational moves and a flip move m→−m to overcome the magnetic anisotropy barrier [27]. At fixed $h^{p}$ and *h*, the obtained MC results were averaged over $N_{conf}$ independent configurations for each BD system. Although simulations were performed for a wide range of polymerization fields, $0\leq h^{p}\leq 100$ kA/m, the three values $h^{p}=20$, 40, and 80 kA/m were considered in more detail. The corresponding values of the Langevin parameters for small and large particles are shown in Table 1.

The static magnetization of the BD system was calculated in the *z* direction of the simulation box as

$$M=\frac{1}{V}{\langle \sum_{i=1}^{N}m_{i}\cos\omega_{i}\rangle }_{t},$$

where ${\langle \ldots \rangle }_{t}$ means the average over simulation time.

The easy axis distribution function $f^{p}$ over the polar angle ξ for the magnetopolymer was calculated using nonuniform set $\xi_{k}=\arccos\left(k/25-1\right)$, for which reverse numbering k=50,49,...,1,0 was applied to avoid a negative sign of $\Delta \xi_{k}=\xi_{k+1}-\xi_{k}$. During the first step, there was a particle number $n_{i}$, for which the value $\cos\xi_{i}$ was in the specified range:

$$f_{p}^{*}\left(\xi_{k}\right)={\langle n_{i}:\cos\xi_{k}\leq \cos\xi_{i}<\cos\xi_{k+1}\rangle }_{N_{conf}},$$

where the angle brackets ${\langle ...\rangle }_{N_{conf}}$ are the average over all saved configurations. The auxiliary function $f_{p}^{*}$ was normalized in the following way:

$$f_{p}\left(\xi_{k}\right)=\frac{f_{p}^{*}\left(\xi_{k}\right)}{\frac{1}{2}\sum_{j=0}^{50}f_{p}^{*}\left(\xi_{j}\right)\sin\xi_{j}\Delta \xi_{j}}.$$

To calculate the second moment $Q_{2}$, the second Legendre polynomial $P_{2}\left(\cos\xi_{i}\right)$ was averaged over all particles and saved magnetopolymer configurations:

$$Q_{2}={\langle \frac{1}{N}\sum_{i=1}^{N}\frac{3\cos^{2}\xi_{i}-1}{2}\rangle }_{N_{conf}}.$$

The radial distribution function *g* characterizes the particle pairs’ number at a given distance *r* in unit volume compared to an ideal gas with equiprobable points of space, where the second particle can be observed [47,48]. A discrete set $\left\{r_{k}\right\}$ was used in the MC simulation, where $r_{k+1}=\Delta r+r_{k}$, $\Delta r=0.025\;d_{s}$, $r_{max}=L/2$. For two types of particle pairs, ij∈{ss,ll}, the calculation of $g_{ij}$ was performed in the direction parallel to the external magnetic field $r||h^{p}$ due to the additional condition $\sqrt{{\left(x_{ij}\right)}^{2}+{\left(y_{ij}\right)}^{2}}\leq h_{0}$, where $h_{0}=10\Delta r$. In common cases, the probability of finding type *j* particle at distance $r_{k}$ from a type *i* i particle in a volume of two cylinders with radius $h_{0}$ and height Δr can be described as

$$g_{ij}^{\left|\right|}\left(r_{k}\right)=\frac{V\times N_{j}\left(r_{k},r_{k}+\Delta r\right)}{2\pi \Delta rh_{0}^{2}\times N_{i}N_{j}}.$$

Such an attempt to calculate the radial distribution function was carried out every 20 MC steps with further averaging of the results over simulation time.

## 5. Discussion

### 5.1. Structural Properties

**Figure 3:**
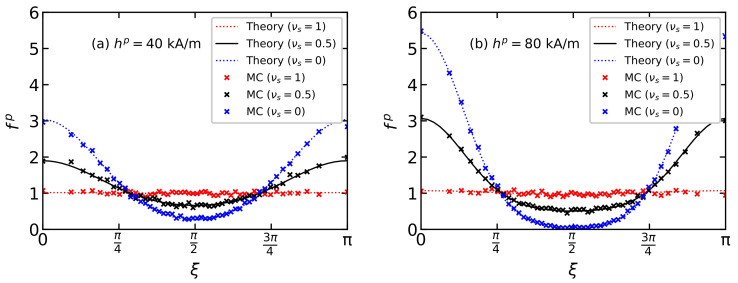
The easy axis distribution function $f_{p}\left(\xi \right)$ over the polar angle ξ for BD systems with a total magnetic volume fraction φ=0.125, placed in the polymerization fields (**a**) $h^{p}=40$ kA/m and (**b**) $h^{p}=80$ kA/m. Parameters of BD configurations are shown in Table 1 and Table 2. Different colors correspond to different values of $\nu_{s}$ as given in the legends. Lines demonstrate the theoretical results from Equations (11) and (12); symbols indicate the MC simulation data.

Figure 3 shows the distribution function of the easy magnetization axes $f_{p}\left(\xi \right)$ in the MPC. The solid lines correspond to Equations (11) and (12); dots denote MC simulation results. Three systems with the same volume concentration of particles φ=0.125 and different fractions of small particles $\nu_{s}=0$0.5, and 1 are considered. The main dimensionless parameters of the systems are presented in Table 1 and Table 2. In a monodisperse system consisting of small particles $\nu_{s}=1$ (red color), all orientations of the easy axes are nearly equiprobable. In a BD system with $\nu_{s}=0.5$ (black color) in comparison with a monodisperse one consisting of large particles $\nu_{s}=0$ (blue color), the probability of an easy axis orientation of a random particle in parallel to the field direction decreases, while the probability of an orientation perpendicular to the field direction increases. These effects are more pronounced with increasing field intensity $h^{p}$, at which the particles were immobilized. It is noteworthy that the intersection of the curves $f^{p}\left(\xi \right)$ for the BD and monodisperse systems occurs at angles $\xi \approx 48^{\circ }$ and $138^{\circ }$. This means that the easy magnetization axis orientation of an arbitrary particle at angles $\xi \approx 48^{\circ }$ and $138^{\circ }$ in the considered monodisperse and BD systems is equiprobable and does not depend on the fractional composition. Theoretical predictions are in good agreement with the MC simulation data.

**Figure 4:**
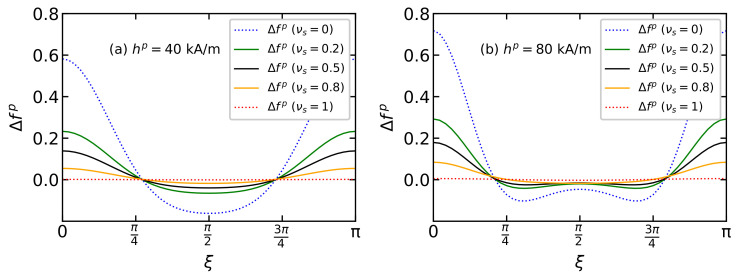
The contribution of dipole–dipole interactions to the easy axis distribution function $\Delta f^{p}\left(\xi \right)=f^{p}\left(\xi \right)-f^{p(id)}\left(\xi \right)$ from Equations (11) and (13) for BD systems with a total magnetic volume fraction φ=0.125, placed in the polymerization field (**a**) $h^{p}=40$ kA/m and (**b**) $h^{p}=80$ kA/m. Parameters of BD configurations are shown in Table 1 and Table 2. Different colors correspond to different values of $\nu_{s}$ as given in the legends.

**Figure 5:**
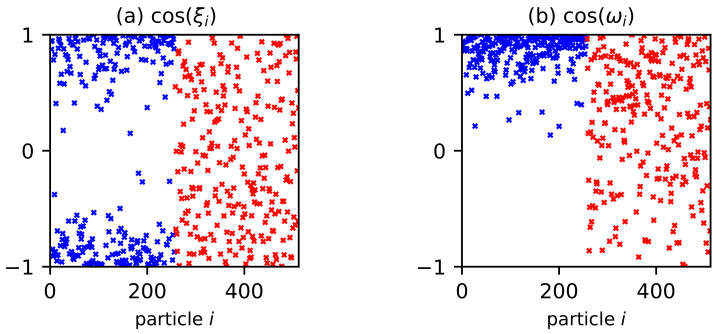
The value of (**a**) $\cos(\xi_{i})$ and (**b**) $\cos(\omega_{i})$ for each particle *i* in a typical BD configuration from MC simulation of the MPC with φ=0.125, $\nu_{s}=0.5$ in an applied magnetic field $h^{p}=80$ kA/m. Parameters of BD configuration are shown in Table 1 and Table 2. Red crosses indicate small particles, and blue crosses indicate large particles.

The contribution of the dipole–dipole interaction to the function of the easy magnetization axes distribution $\Delta f^{p}\left(\xi \right)=f^{p}\left(\xi \right)-f^{p(id)}\left(\xi \right)$ is determined from Equations (11)–(13), as shown in Figure 4. Dipole–dipole interactions increase the probability of easy axes orientation parallel to the field (ξ=0 and ξ=π) while decreasing the probabilityof an orientation perpendicular to the field (ξ=π/2). This behavior is explained as follows. The easy magnetization axis ${\hat{n}}_{i}$ is the energetically favorable position of the magnetic moment inside the particle: the orientation of the magnetic moment ${\hat{m}}_{i}$ in the directions of the vectors ${\hat{n}}_{i}$ and $-{\hat{n}}_{i}$ is equiprobable. Due to dipole–dipole interactions, it is energetically preferable for magnetic moments to form nose-to-tail structures. In a system of moving particles, the magnetic moment, rotating together with the body of the particle, “pulls” the easy axis along with it. In an external magnetic field, the magnetic moments of the particles tend to align with the direction of the field, and the nose-to-tail dipolar structures react to the field more significantly than single particles. Thus, an anisotropic texture of easy axes is formed in the system with a preferred parallel alignment to the applied field. This texture is retained at polymerization. The formation of an anisotropic texture of the easy magnetization axes is clearly visible in computer simulations (Figure 5a). The alignment of the easy magnetization axes parallel to the field occurs mainly due to large particles (blue dots), which interact more strongly with each other and react more significantly to the external field $h^{p}$. Figure 5b shows a snapshot of the magnetic moment directions in the same system. A preferred direction of magnetic moments along the field $h^{p}$ is observed; this behavior is most pronounced for the large particles.

**Figure 6:**
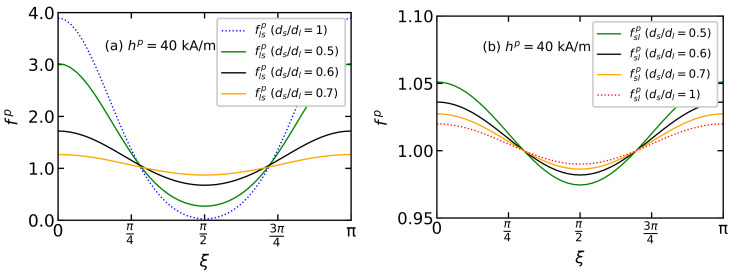
The easy axis distribution function of (**a**) large $f_{ls}^{p}\left(\xi \right)$ and (**b**) small particles $f_{sl}^{p}\left(\xi \right)$ over the polar angle ξ for BD system with volume φ=0.3, small particle fraction $\nu_{s}=0.8$ and $h^{p}=40$ kA/m. Different colors correspond to different relation of diameters $d_{s}/d_{l}$ as given in the legends. Lines represent the theoretical results from Equations (12).

**Figure 7:**
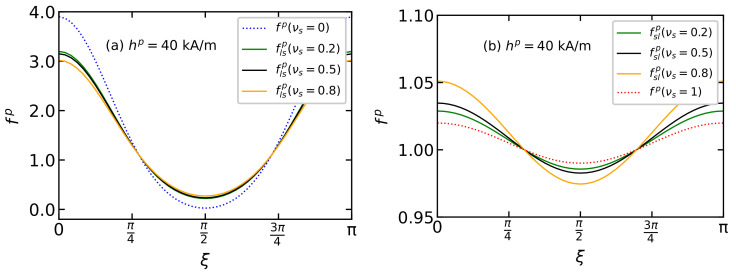
The easy axis distribution function of (**a**) large $f_{ls}^{p}\left(\xi \right)$ and (**b**) small particles $f_{sl}^{p}\left(\xi \right)$ over the polar angle ξ from Equation (12) for BD systems with the total magnetic volume fraction φ=0.3, placed in the polymerization field $h^{p}=40$ kA/m. Parameters of BD configurations are shown in Table 1 and Table 2. Different colors correspond to different values of $\nu_{s}$ as given in the legends.

The influence of particles of one fraction on the orientation of the easy magnetization axes of particles of another fraction in a BD system is shown in Figure 6 and Figure 7. Dashed lines denote monodisperse systems consisting only of large $d_{s}=10$ nm (blue color) or small $d_{s}=5$ nm (red color) particles, while solid lines correspond to the distribution functions of easy axes of large $f_{ls}^{p}\left(\xi \right)$ and small $f_{sl}^{p}\left(\xi \right)$ particles in a BD system, which are determined from Equation (12). The volume concentration of the monodisperse and BD systems is the same φ=0.3. Figure 6 demonstrates the influence of the size of large particles on the texturing of the easy magnetization axes of a BD system with $\nu_{s}=0.8$ and $d_{s}=5$ nm. An increase in the diameter of large particles in a BD system leads to an increase in the probability of orientation of the easy axes of large particles parallel to the field and a decrease in the probability of their orientation perpendicular to the field. (Figure 6a). This indicates the orientational ordering of large particles with an increase in their size. It should be noted that the addition of small particles to a monodisperse system containing only large particles $d_{s}=10$ nm disorders the texturing of the easy magnetization axes of large particles. The opposite trend is observed when large particles are added to a monodisperse system of small particles (Figure 6b). In this case, the appearance of large particles in the system leads to the ordering of the easy magnetization axes of small particles; the larger the particle size in the large fraction, the more small particles there are with their easy axes oriented parallel to the field in the system. Thus, an increase in the particle size of the large fraction leads to the ordering of the easy magnetization axes of both large and small particles.

**Figure 8:**
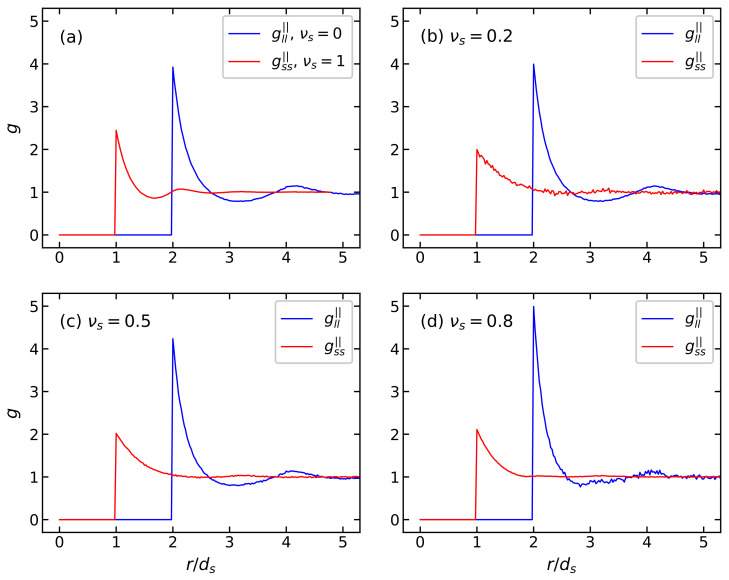
The radial distribution function $g^{\left|\right|}$ for BD systems with φ=0.3 in the parallel direction of the external magnetic field $h^{p}=40$ kA/m: (**a**) monodisperse systems, (**b**) $\nu_{s}=0.2$, (**c**) $\nu_{s}=0.5$, and (**d**) $\nu_{s}=0.8$. Parameters of BD configurations are shown in Table 1 and Table 2. Red lines correspond to MC results for the small particle fraction, blue lines indicate MC data for thelarge particle fraction.

Figure 7 shows the orientation distribution function of the easy magnetization axes of large $f_{ls}^{p}\left(\xi \right)$ and small $f_{sl}^{p}\left(\xi \right)$ particles for a BD system with $d_{s}/d_{l}=0.5$ for different proportions of small particles $\nu_{s}$. On the one hand, the presence of small particles in the system contributes to the disordering of large particles: with an increase in the concentration of small particles, the probability $f_{ls}^{p}\left(\xi \right)$ of easy axes orientation of the large particles along the direction of the field decreases (Figure 7a). On the other hand, the addition of large particles to a monodisperse system of small particles leads to the organization of small particles, increasing the probability $f_{sl}^{p}\left(\xi \right)$ of orientation of their easy magnetization axes along the direction of the field and decreasing the probability of orientation perpendicular to the field (Figure 7b). However, with an increase in the number of large particles in a BD system, a texture disorder in the small particle easy axes is observed. This unexpected behavior is explained by the specifics of the model under consideration: a change in the dispersal composition leads to a change in the system’s volume since the total volume concentration, the total number of particles and the size of the particles remain constant. Thus, with an increase in the number of large particles in a BD system, the average distance between particles increases, which leads to a weakening of interparticle interactions and, as a consequence, to disordering of the texturing of the easy magnetization axes of small particles. The disordering of particles of a small fraction in a BD system with an increase in the concentration of large particles is clearly seen in the radial distribution functions (Figure 8) determined using MC simulation. In a monodisperse system of small particles, the radial distribution function clearly shows a local maximum in the region $r=2d_{s}$ (Figure 8a, red curve), demonstrating a long-range order, which arises due to long-range dipole–dipole interactions. When large particles are added to the system, this maximum is smoothed out, and the maximum in the region $r=d_{s}$ decreases, which indicates a weakening of the interactions between small particles. At the same time, the appearance of particles of the small fraction in a monodisperse system of large particles leads to an increase in the maximum of the radial distribution function of large particles (Figure 8a, blue curve) in the region $r=d_{s}$. This means that the probability of detecting a “large-large” dimer increases with an increase in the concentration of small particles in a BD system.

**Figure 9:**
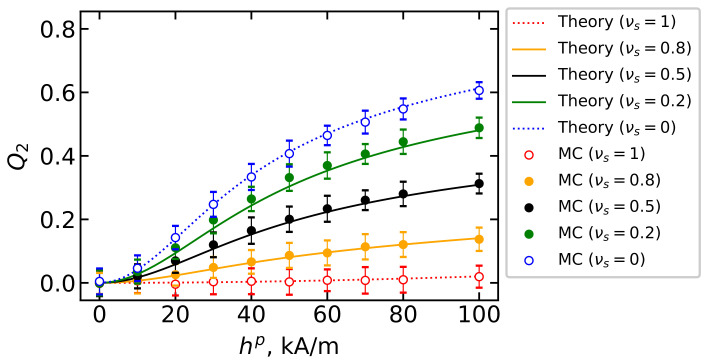
The second moment $Q_{2}$ of easy axis orientation over the polymerization field $h^{p}$ for BD systems with the total magnetic volume fraction φ=0.125. Parameters of BD configurations are shown in Table 1 and Table 2. Different colors correspond to different values of $\nu_{s}$ as given in the legend. Lines demonstrate the theoretical results from Equation (14), symbols indicate the MC simulation data.

Figure 9 shows the second moment for the easy axes of an arbitrary particle in BD systems with different parts of large and small particles and a constant volume concentration φ=0.125 depending on the intensity of the field $h^{p}$ in which polymerization occurs. The theory from Equation (14) (curves) is shown in comparison with the computer simulation results (dots). There is good agreement between the data. An increase in the intensity of the field at which the immobilization of particles occurs promotes the ordering of the axes of easy magnetization of particles in all systems under consideration: in weak fields ($h^{p}≲10$ kA/m), the value of *Q*_2_ is close to zero, which indicates the absence of texturing for the easy axes in all samples; in strong fields ($h^{p}\approx 100$ kA/m), a wide variety of *Q*_2_ values is observed, the greatest texturing of which occurs in a system with a large number of large particles. Thus, by changing the dispersion composition of the sample at a constant volume concentration of magnetic filler particles, one can control the sample’s orientational texturing.

### 5.2. Magnetic Properties

**Figure 10:**
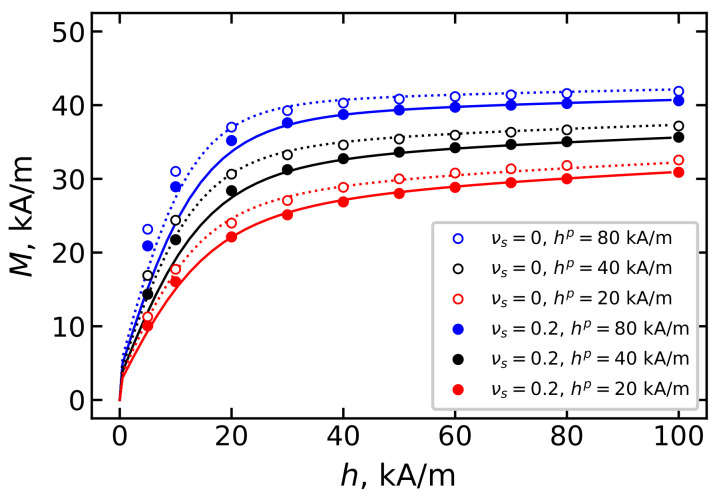
MCP magnetization *M* over the applied magnetic field *h* for BD systems with the total magnetic volume concentration φ=0.125. Parameters of BD configurations are shown in Table 1 and Table 2. Lines demonstrate the theoretical results from Equation (16), symbols indicate the MC simulation data. Dotted lines and unfilled symbols represent the case of $\nu_{s}=0$; dashed lines and filled symbols show the result for $\nu_{s}=0.2$. Different colors correspond to different values of $h^{p}$ as given in the legends.

The orientation texture of the easy axes formed during synthesis further determines the sample’s response to an external magnetic field. Figure 10 shows the magnetization of an ensemble of immobilized magnetic particles in a BD system with $\nu_{s}=0.2$ (solid lines and filled symbols) in comparison with a monodisperse system (dotted lines and unfilled symbols). It is clear that samples with a strongly pronounced orientation texture, which, as shown in the previous section, is formed at large values of the $h^{p}$ field, are better magnetized. Consequently, by adjusting the intensity of the field $h^{p}$ applied to the sample during polymerization, it is possible to synthesize magnetopolymer composites with a predetermined magnetization.

**Figure 11:**
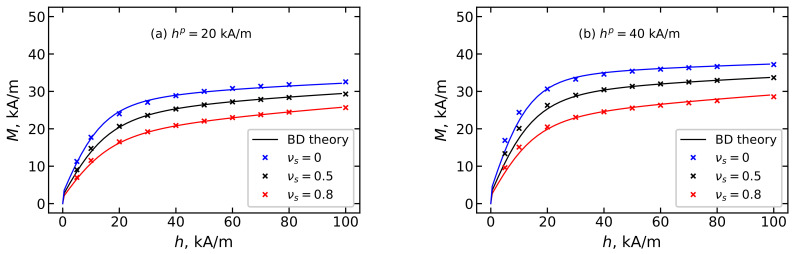
MCP magnetization *M* with BD magnetic filler in the applied magnetic field *h*. The total volume concentration of magnetic filler is φ=0.125; different colors correspond to different values of $\nu_{s}$. Parameters of BD configurations are shown in Table 1 and Table 2. MCP is obtained by polymerization in the field (**a**) $h^{p}=20$ kA/m and (**b**) $h^{p}=40$ kA/m. Lines demonstrate the theoretical results from Equation (16), and symbols indicate the MC simulation data.

Another way to control the magnetization of the MPC is to change the dispersion composition. Figure 11 shows the magnetization of an ensemble of immobilized particles with a volume concentration of φ=0.125 and with a different fraction of small particles. Samples containing more small particles show less magnetization. In samples where the immobilization of particles occurs in the field $h^{p}=40$ kA/m (Figure 11b), the magnetization is more sensitive to changes in the dispersion composition than in samples polymerized in the field $h^{p}=20$ kA/m (Figure 11a). It is worth noting that the theoretical prediction of magnetization obtained from Equations (16) and (17), and represented in Figure 10 and Figure 11, agrees well with the results of MC simulation.

**Figure 12:**
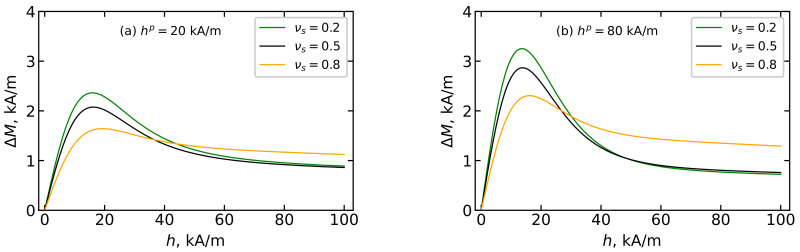
Theoretical data of the contribution of dipole–dipole interactions to MPC magnetization $\Delta M=M-M_{id}$ over the applied magnetic field *h* for BD systems with the total magnetic volume fraction φ=0.125, obtained in the polymerization field (**a**) $h^{p}=20$ kA/m and (**b**) $h^{p}=80$ kA/m. Parameters of BD configurations are shown in Table 1 and Table 2. Different colors correspond to different values of $\nu_{s}$ as given in the legends.

Special attention should be paid to the contribution of the dipole–dipole interaction $\Delta M=M-M^{\left(id\right)}$ to the magnetization of ensembles of immobilized particles, which is shown in Figure 12. The maximum value of ΔM is observed at h≈18 kA/m, and the position of the maximum weakly depends on the value of $h^{p}$; with the growth of $h^{p}$, the value of the maximum of ΔM increases. The latter indicates that dipole–dipole interactions manifest themselves more strongly in a system that has been textured in a larger field $h^{p}$. The decrease of ΔM indicates that magnetic moment-field interactions begin to prevail over the dipole–dipole interaction. However, even at h≈100 kA/m, ΔM is not equal to zero, which indicates the influence of dipole–dipole interactions on the magnetization of the considering system at any intensity of applied magnetic field including sufficiently strong fields.

**Figure 13:**
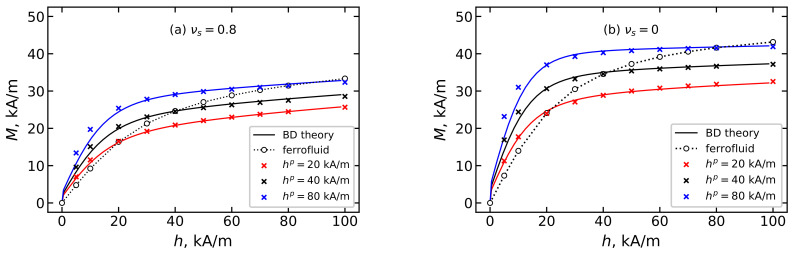
Comparison of magnetization of the MCP and ferrofluid with the same dispersion composition. Total magnetic particle volume concentration φ=0.125 and value of small particles’ fraction: (**a**) $\nu_{s}=0.8$ and (**b**) $\nu_{s}=0$. Parameters of BD configurations are shown in Table 1 and Table 2. Solid lines demonstrate the theoretical results from Equations (16), symbols indicate the MC simulation data. Unfilled points show the simulation results of ferrofluid magnetization before polymerization, while crosses represent the MC data for the MPC. Different colors correspond to different values of the polymerization field $h^{p}$ as given in the legends.

Figure 13 shows the magnetization of the MPC and the dispersion of magnetic particles in a liquid carrier (ferrofluid). In all samples, the volume concentration of the magnetic filler is φ=0.125. To estimate the effect of polydispersity on the magnetization of samples, a BD system with a fraction of small particles $\nu_{s}=0.8$ (Figure 13a) is shown in comparison with a monodisperse system of large particles $\nu_{s}=0$ (Figure 13b). In weak fields, the MPC is magnetized better than the ferrofluid, since the orientation texture formed in the MPC promotes its magnetization. At $h=h^{p}$, the magnetization of the MPC and ferrofluid coincide. This happens because the MPC “remembers” the microstructures of the ferrofluid at the moment of particle immobilization and demonstrates behavior typical for a ferrofluid. At $h>h^{p}$, the magnetization of the MPC is less than that of the ferrofluid. This behavior is explained by the fact that in order to magnetize the MPC in field $h>h_{p}$, the magnetic moments of the immobilized particles, tending to align with the direction of the field, must deviate from the easy axes, which is hindered by the internal magnetocrystallographic anisotropy of the particles, the value of which is determined by the parameter σ. From Figure 13, it is clear that the dispersion composition of the ferrofluid on the basis of which the MCP will be formed, as well as the conditions under which the polymerization of the carrier liquid occurs, are tools for adjusting the magnetic properties of the MCP.

## 6. Conclusions and Outlook

In this paper, a theory of the magnetic response of MPCs with BD magnetic fillers to a static magnetic field has been developed. The obtained analytical expressions make it possible to calculate the magnetization and distribution function of the easy axes of magnetic filler particles depending on the dispersion composition, the concentration of magnetic particles, the intensity of dipole–dipole interactions, and sample synthesis conditions. It has been shown that the dispersion composition and the intensity of the polymerization field are the parameters that make it possible to control MPC magnetization. Based on the obtained analytical regularities, the chain between the MPC synthesis conditions, the magnetic filler texture formed, and the MPC properties were traced. A deep understanding of the features of this multi-parameter and multi-conditional chain is the basis for designing MPCs with a predetermined magnetic response to an external magnetic field. Further work on systematic testing of theoretical results on experimental data is in progress.

## Figures and Tables

**Table 1:** Characteristics of small and large particles in a BD system. The values of the diameters $d_{i}$, the anisotropy parameters $\sigma_{i}$, the dipolar coupling constant $\lambda_{i}$, and the Langevin parameters $\alpha_{i}^{p}$ for both small and large particles are calculated at room temperature T=293 K for CoFe_2_O_4_.

Fraction	$d_{i}$, nm	$\sigma_{i}$	$\lambda_{i}$	$\alpha_{i}^{p}$ (20 kA/m)	$\alpha_{i}^{p}$ (40 kA/m)	$\alpha_{i}^{p}$ (80 kA/m)
Small	5	3.236	0.1355	0.1627	0.3253	0.6506
Large	10	25.887	1.0843	1.3012	2.6024	5.2048

**Table 2:** Dimensionless parameters of BD systems with volume concentrations φ=0.125 and φ=0.3. Values of the particle numbers $N_{i}$, the volume concentrations $\varphi_{i}$, the Langevin susceptibilities $\chi_{L}^{i}$ and the computer simulation box side *L* for both types of particles at different fractions $\nu_{s}$. In all cases, the value of *L* is dimensionless at $d_{s}$.

	Parameter	$\nu_{s}=1$	$\nu_{s}=0.8$	$\nu_{s}=0.5$	$\nu_{s}=0.2$	$\nu_{s}=0$
N=512	$N_{s}$	512	410	256	102	0
	$N_{l}$	0	102	256	410	512
φ=0.125	$\varphi_{s}$	0.125	0.0418	0.0139	0.0038	0.000
	$\varphi_{l}$	0.000	0.0832	0.1111	0.1212	0.125
	$\chi_{L}^{s}$	0.1355	0.0452	0.0151	0.0041	0.000
	$\chi_{L}^{l}$	0.000	0.7229	0.9639	1.0515	1.08
	*L*	12.896	17.253	21.291	24.197	25.792
φ=0.300	$\varphi_{s}$	0.300	0.1003	0.0333	0.0090	0.000
	$\varphi_{l}$	0.000	0.1997	0.2667	0.2910	0.300
	$\chi_{L}^{s}$	0.325	0.1087	0.036	0.0098	0.000
	$\chi_{L}^{l}$	0.000	1.732	2.3132	2.524	2.602
	*L*	9.632	12.886	15.902	18.072	19.264

## Data Availability

The data presented in this study are available on request from the corresponding author.

## Abbreviations

The following abbreviations are used in this manuscript:

MPC	Magnetopolymer composite
MC	Monte Carlo
BD	Bidisperse
